# Effect of substituting fresh‐cut perennial ryegrass with fresh‐cut white clover on bovine milk fatty acid profile

**DOI:** 10.1002/jsfa.8991

**Published:** 2018-04-15

**Authors:** Sokratis Stergiadis, Deborah N Hynes, Anna L Thomson, Kirsty E Kliem, Carolina GB Berlitz, Mevlüt Günal, Tianhai Yan

**Affiliations:** ^1^ Animal, Dairy and Food Chain Sciences, School of Agriculture, Policy and Development University of Reading Reading UK; ^2^ Agriculture Branch, Sustainable Agri‐Food Sciences Division Agri‐Food and Biosciences Institute Hillsborough, Co. Down UK; ^3^ Department of Animal Science Federal University of Rio Grande do Sul Porto Alegre RS Brazil; ^4^ Department of Animal Science Süleyman Demirel University Isparta Turkey

**Keywords:** white clover, fresh‐cut grass, dairy cows, milk fatty acids, transfer efficiency

## Abstract

**BACKGROUND:**

Including forage legumes in dairy systems can help address increasing environmental/economic concerns about perennial ryegrass monoculture pastures. This work investigated the effect of substituting fresh‐cut grass with increasing quantities of fresh‐cut white clover (WC) on milk fatty acid (FA) profile and transfer efficiency of dietary linoleic (LA) and α‐linolenic (ALNA) acids to milk fat. Three groups of three crossbred dairy cows were used in a 3 × 3 crossover design. Dietary treatments were 0 g kg^−1^ WC + 600 g kg^−1^ grass, 200 g kg^−1^ WC + 400 g kg^−1^ grass, and 400 g kg^−1^ WC + 200 g kg^−1^ grass. All treatments were supplemented with 400 g kg^−1^ concentrates on a dry matter basis. Cows had a 19‐day adaptation period to the experimental diet before a 6‐day measurement period in individual tie stalls.

**RESULTS:**

Increasing dietary WC did not affect dry matter intake, milk yield or milk concentrations of fat, protein or lactose. Milk polyunsaturated FA concentrations (total n‐3, total n‐6, LA and ALNA) and transfer efficiency of LA and ALNA were increased with increasing dietary WC supply.

**CONCLUSION:**

Inclusion of WC in pastures may increase concentrations of nutritionally beneficial FA, without influencing milk yield and basic composition, but any implications on human health cannot be drawn. © 2018 The Authors. *Journal of the Science of Food and Agriculture* published by JohnWiley & Sons Ltd on behalf of Society of Chemical Industry.

## INTRODUCTION

Pasture‐based livestock systems are essential to human nutrition and global food security because of ruminants' ability to transform material that is inedible to humans (forage plants) into highly nutritious human food (milk and meat).^1^ However, the over‐reliance on perennial ryegrass pastures in pasture‐based systems with high stocking rates requires substantial input of inorganic nitrogen (N) fertilizers to maintain increased dry matter (DM) yields per hectare throughout the grazing season.^2^ This may contribute to substantial groundwater and air pollution.^3^, ^4^ Restrictions to N fertilization have been legislated in the EU and UK,^3^, ^5^ but these pose a risk to future grass productivity and economic viability of dairy farms.^6^ Subsequently, there is a renewed interest in forage legumes, which were widely used prior to the introduction of mineral N fertilizer, as they demonstrate unique nutritional, environmental and economic advantages and high adaptability in various climates.^2^, ^7^, ^8^ Using forage legumes also reduces the reliance on imported high‐protein feeds and N fertilizer, because they are rich in protein and fix atmospheric N.^7^, ^8^, ^9^ However, bloat control measures via on‐farm management and/or plant breeding may be required to prevent excessive consumption of bloat‐inducing legumes.^7^, ^9^ In the UK, the most commonly recommended legume for grazing systems is white clover (WC; *Trifolium repens* L.).^10^ Dairy cows on WC‐containing pastures have shown higher productivity^11^, ^12^ than cows fed on grass pastures, although the beneficial effect on productivity has not been consistent across the studies^13^, ^14^ and a decrease in milk fat concentrations has been observed on some occasions.^12^, ^15^

Further interest in including WC in dairy cow diets has emerged after studies reported that grazing WC may improve milk fatty acid (FA) profile,^15^ which is of benefit to consumers.^16^, ^17^, ^18^ Over the last 40 years, and with the increasing incidence of cardiovascular disease (CVD), obesity and cancer, consumers have become more aware of the saturated FA (SFA) content of milk fat,^16^, ^17^ as some SFA (C12:0, C14:0 and C16:0) can increase blood levels of low‐density lipoprotein cholesterol.^17^ However, milk fat also contains monounsaturated FA (MUFA), such as *c*9 C18:1 (OA, oleic acid) and *t*11 C18:1 (VA, vaccenic acid), and polyunsaturated FA (PUFA), such as *c*9*t*11 C18:2 (RA, rumenic acid) and the omega‐3 (n‐3) *c*9*c*12*c*15 C18:3 (ALNA, α‐linolenic acid), *c*5*c*8*c*11*c*14*c*17 C20:5 (EPA, eicosapentaenoic acid) and *c*7*c*10*c*13*c*16*c*19 C22:5 (DPA, docosapentaenoic acid), which have been associated with a number of benefits for human health.^17^, ^19^, ^20^, ^21^, ^22^, ^23^ These include (i) reductions in blood concentrations of low‐density lipoprotein cholesterol and triacylglycerol and the risk of CVD (when replacing SFA in human diets), (ii) action against dislipidaemia and cancer, (iii) protective role against fracture risk, type 2 diabetes, obesity, inflammation and oxidation of membrane cells, (iv) development and/or improvement of neuronal, retinal and immune function in fetuses, and (v) enhancement of cognitive, brain and immune functions and bone health.^17^, ^19^, ^20^, ^21^, ^22^, ^23^

In previous studies, cows grazing grass/WC swards produced milk with higher concentrations of total n‐3 PUFA, including ALNA, when compared with milk from cows grazing grass swards.^15^ However, under grazing conditions, measuring individual cow DM intake (DMI) and collecting representative samples of individual cow feed is challenging,^24^ thus making it difficult to accurately calculate transfer efficiency of dietary LA and ALNA to milk. In addition, there are difficulties in maintaining a constant ratio of grass/WC intake at pasture due to the highly variable contribution of each forage in the sward throughout the season,^25^ as well as the potential selective grazing from cows. To our knowledge, there are no published studies investigating the effect of substituting fresh‐cut grass with fresh‐cut WC on milk FA profiles and transfer efficiencies of dietary LA and ALNA to milk. Such a study would enhance knowledge in this area and provide potential explanations to the underlying mechanisms of the effect of WC. The main objective of this study was therefore to evaluate the effect of fresh‐cut WC inclusion (at different rates) in a fresh‐cut grass‐based diet on cow's milk FA profiles and the transfer efficiency of dietary LA and ALNA to milk, under a constant forage:concentrate ratio.

## MATERIALS AND METHODS

The present study was performed according to the regulations of the Department of Health, Social Services and Public Safety of Northern Ireland, in line with the Animal (Scientific Procedures) Act 1986.^26^

### Experimental design

This study was conducted at Agri‐Food and Biosciences Institute, Hillsborough, UK, between 8 June 2015 and 2 September 2015, using nine multiparous lactating crossbred (Holstein × Swedish Red) cows of (mean ± SE) 30 ± 4.4 kg d^−1^ milk yield, 522 ± 39.2 kg live weight and 92 ± 34.6 days in milk, blocked into three groups according to these parameters. The experimental design was a 3 × 3 crossover design, consisting of three experimental periods of 25 days, so that all groups of three cows consumed each of the three dietary treatments. Diets had a constant forage:concentrate ratio of 60:40 (on a DM basis), which is a common forage:concentrate ratio used in dairy diets in Northern Ireland, and ensured that cows had adequate energy to produce according to their genetic merit.^27^ Dietary treatments, on a DM basis, were: 0 g kg^−1^ WC + 600 g kg^−1^ perennial ryegrass (Control), 200 g kg^−1^ WC + 400 g kg^−1^ perennial ryegrass (Low‐WC), and 400 g kg^−1^ WC + 200 g kg^−1^ perennial ryegrass (High‐WC). All diets were supplemented with 400 g kg^−1^ concentrates. Each experimental period consisted of a group‐housed adaptation phase (18 days) whereby measurements of daily individual feed intakes were recorded, an adaptation phase in individual tie‐stalls (1 day) and a measurement phase in individual tie‐stalls (6 days).^28^

### Experimental diets and feeding

Pure perennial ryegrass swards and pure clover swards were zero‐grazed throughout the experiment to maximize the control over the proportions of each forage intake in cow diets. The grass sward was originally sown 4 years prior to the commencement of the experiment with the varieties Aberstar and Aberzest, and the WC cultivar Alice, at a seed density ratio of 8:5:1. However, continuous N fertilization at an average annual level of 175 kg N ha^−1^ (and P and K according to requirements) over 4 years resulted in grass outcompeting WC by the time the experiment commenced. Therefore, grass represented, on average, 977 g kg^−1^ of the sward biomass throughout the experiment. The grass sward had not been grazed the year prior to the experiment, and was zero‐grazed in May in order to create plots with staggered growth. The grass sward was fertilized with 40 kg N ha^−1^ within 3 days post harvest on four occasions (one pre‐experimental cut and three cuts during the experiment), using a Vicon SuperFlow spreader (Kverneland Group UK Ltd, Merseyside, UK). This resulted in an annual fertilisation rate of 160 kg N ha^−1^.

For the establishment of the pure WC sward, the variety Aran was used, which is considered the most productive WC variety among the recommended WC varieties for Northern Ireland,^29^ and its large leaf area makes it appropriate for the zero‐grazing practices in the current study. Establishment of the WC sward took place the year before commencement of the trial. In the sowing year, the sward was (i) grazed by sheep in early March, and then treated with glyphosate‐containing herbicides (Roundup™; Monsanto Technology LLC, OH, USA) at 25% strength,^30^ to inhibit grass growth, (ii) sown in late March solely with WC (variety Aran) at a rate of 8 kg seed ha^−1^, (iii) fertilized at 40 kg P ha^−1^ and 40 kg K ha^−1^ using a Vicon SuperFlow spreader (Kverneland Group UK Ltd, Merseyside, UK) in April, and (iv) zero‐grazed in July and September. In the year of the experiment, the WC sward was fertilized in April at 40 kg P ha^−1^ using a Vicon SuperFlow spreader, and zero‐grazed in May, in order to create plots with staggered growth. Throughout the experiment the WC sward contained >977 g kg^−1^ of the biomass as WC.

Fresh‐cut forage from the grass or WC swards was harvested daily at 1000 am using a zero‐grazer (GT80, Future Grass Technology Ltd, Ireland). It was immediately boxed loosely in plastic containers holding approximately 10–12 kg of fresh‐cut forage and kept at ambient temperature, in well‐ventilated areas of the barn, so that potential wilting during storage was minimized. Fresh‐cut forage was delivered to feeders in several batches throughout the day so that cows had constant free access to it for 23 h d^−1^. Grass and WC were harvested at regrowth intervals of 23–30 and 33–40 days, respectively, in order to mimic typical rotation grazing strategies and ensure that forage of similar stage of growth was fed across the study. For each cow, *ad libitum* fresh‐cut forage intake was monitored for the initial 7 days of each experimental period, by providing access to individual automatic feeders activated by neck sensors; subsequently, fresh‐cut forage was offered at 105% *ad libitum* intake. For the Low‐WC and High‐WC diets, grass and WC were mixed thoroughly by hand and offered in the same feeder.

Concentrates were offered as two equal meals during milking at 0700 h and 1500 h. These were offered within plastic trays, placed on top of the forage in the feeders, thus avoiding mixing forage and concentrates and enabling the measurement of *ad libitum* fresh‐cut forage intake. The same concentrate feed ingredients were fed across all experimental treatments, with slight variations in the offered quantities of each ingredient, to meet energy and protein requirements of the cows, provide iso‐nitrogenous diets and maintain a constant forage:concentrate ratio of 60:40 (on a DM basis), across all treatments. The chemical composition of the three diets is presented in Table 1. Allocation of concentrates was undertaken in three steps. First, real‐time assessments of forage DM, to be used in the calculations of daily forage allocation, were performed daily by microwaving at full power (700 W) until constant weight (Sanyo microwave oven, Osaka, Japan).^31^ Second, the supply of crude protein from forage was evaluated by daily real‐time assessments for grass, using near‐infrared spectroscopy (using an NIRS™ 5000/6500 Feed and Forage analyser; Foss, Hillerod, Denmark), and twice‐a‐week real‐time assessments of WC crude protein, using the Dumas combustion method (using a Vario Max CN analyser; Elementar, Hanau, Germany). The first two steps enabled calculation of the expected protein intake from forage according to the average feed intake and forage protein content over the previous 3 days. Thirdly, three types of concentrate pellets with contrasting crude protein contents, made from different concentrations of similar ingredients, were combined as required to meet energy and protein requirements of the cows, and provide iso‐nitrogenous diets at a constant forage:concentrate ratio of 60:40 (on a DM basis). This practice minimized the potentially confounding effect of concentrate feed, especially on dietary FA supply, between the different treatments. Cows had free access to water throughout the study.

**Table 1:** Chemical composition and fatty acid profiles of the total diet offered to cows over the three experimental periods of the feeding trial

Parameters assessed	Dietary treatment^a^		
	Control	Low‐WC	High‐WC
DM (g kg^−1^ fresh weight)	465	441	432
Organic matter (g kg^−1^ DM)	931	915	910
Crude protein (g kg^−1^ DM)	186	193	194
Gross energy (MJ kg^−1^ DM)	18.6	18.6	18.3
Neutral‐detergent fibre (g kg^−1^ DM)	416	390	362
Acid‐detergent fibre (g kg^−1^ DM)	203	200	193
Water‐soluble carbohydrates (g kg^−1^ DM)	93	69	51
Starch (g kg^−1^ DM)	107	119	138
Fatty acid profiles (g kg^−1^ DM)			
C16:0	5.8	6.2	6.0
C18:0	0.6	0.6	0.7
OA	3.2	3.1	2.8
LA	3.1	3.2	3.3
ALNA	6.3	6.9	7.1
Total fatty acids	21.0	22.4	21.9

DM, dry matter; WC, white clover; OA, oleic acid; LA, linoleic acid; ALNA, α‐linolenic acid.

a Control, 0 g kg^−1^ fresh‐cut WC + 600 g kg^−1^ fresh‐cut grass + 400 g kg^−1^ concentrate; Low‐WC, 200 g kg^−1^ fresh‐cut WC + 400 g kg^−1^ fresh‐cut grass + 400 g kg^−1^ concentrate; High‐WC, 400 g kg^−1^ fresh‐cut WC + 200 g kg^−1^ fresh‐cut grass + 400 g kg^−1^ concentrate (on a DM basis).

### Measurements

During the measurement phase, cows were kept in individual tie‐stalls that allowed accurate recording of feed intakes, which were calculated as the difference between the pre‐weighed feed offered in the individual feeders and refusals the following day. Fresh‐cut grass and WC samples were collected daily and analysed daily for oven DM at 85 °C (until constant weight) during the 6‐day measurement phase. Fresh‐cut forage samples were oven‐dried at 60 °C for 48 h, milled through a 0.8 mm screen and analysed, by wet chemistry, for contents of N, gross energy (GE),^32^ water‐soluble carbohydrates (WSC),^33^ acid‐detergent fibre (ADF), neutral‐detergent fibre (NDF)^34^ and ash.^35^ Concentrate samples (200 g) were collected four times per week and dried for 48 h at 100 °C. They were composited into one sample, milled through a 0.8 mm screen and analysed, by wet chemistry, for weekly determination of DM, N, GE, WSC, ADF, NDF, ash (using the same methods as forage) and starch.^36^ Dry forage samples (composited for each 6‐day measurement phase) and concentrate samples (composited for each experimental period) were analysed for FA content and profiles according to the methods described by Kliem *et al*.^37^ The FA profiles of the three diets are presented in Table 1.

Milk yield was recorded automatically during milking using portable milking machines (Waikato Milking Systems NZ Ltd, Hamilton, New Zealand) in the individual tie‐stalls. Milk samples at 2% of total volume were also collected daily (morning and afternoon), and a composite sample for each 6‐day measurement phase was used for analysis. These milk samples were analysed for concentrations of fat, protein and lactose, using a Milkoscan FT6000 (Foss). Milk FA profiles were analysed by gas chromatography (Bruker 350 GC, Bruker, Germany) using previously published esterification and methylation methods, and identification and quantification techniques.^38^ Carbon deficiency in the flame ionization detector response for FA methyl esters containing between 4 and 10 atoms of carbon was accounted for, using a combined correction factor, as previously described.^39^

### Statistical analysis

All statistical analyses were performed using GenStat.^40^ Analysis of variance (ANOVA), derived from linear mixed‐effects models (residual maximum likelihood analysis),^41^ by considering (i) dietary treatment (Control, 0 g kg^−1^; Low‐WC, 200 g kg^−1^; High‐WC, 400 g kg^−1^ fresh‐cut WC in offered DM) and experimental period (1st, 2nd, 3rd) as fixed factors and (ii) cow and its start date in the measurement phase as random factors. A significant effect of treatment was declared when *P* < 0.05 and tendencies were declared when 0.05 < *P* < 0.10. The residual diagnostics of the final model were assessed using normality plots, with no data showing deviation from normality. Pairwise comparisons of means (*P* < 0.05) were performed using Fisher's least significant difference test.

Atherogenicity index (AI) and thrombogenicity index (TI), as markers to indicate potential risk of CVD, were calculated based on milk FA profile and according to Srednicka‐Tober *et al*.,^42^ as follows:

$$\mathrm{AI}=\left(C12:0+4\times C14:0+C16:0\right)/\left(\text{MUFA}+\text{PUFA}\right)$$

$$\begin{array}{cc} & \mathrm{TI}=\left(C14:0+C16:0+C18:0\right)/\left[\left(0.5\times \text{MUFA}\right)\right.\\ & \left.+\left(0.5\times n-6\right)+\left(3\times n-3\right)+\left(n-3/n-6\right)\right] \end{array}$$

Δ^9^‐Desaturase activity index (Δ^9^I) was calculated based on milk FA profile and according to Kay *et al*.,^43^ as follows:

$$\begin{array}{cc} & \Delta^{9}I=\left(c9\;C14:1+c9\;C16:1+\mathrm{OA}+\mathrm{RA}\right)/\left(c9\;C14:1+\right.\\ & \left.c9\;C16:1+\mathrm{OA}+\mathrm{RA}+C14:0+C16:0+C18:0+\mathrm{VA}\right) \end{array}$$

Transfer efficiency of dietary *c*9*c*12 C18:2 (LA, linoleic acid) and ALNA from feed to milk was calculated according to Stergiadis *et al*.,^44^ as LA/ALNA in milk (g) / LA/ALNA intake (g), where:

- LA/ALNA in milk (g) = milk yield (g) × [milk fat content (g kg^−1^ milk) / 1000)] × [LA/ALNA (g kg^−1^ total FA) × 0.933 / 1000)], with 0.933 representing % FA in total milk fat;^45^
- LA/ALNA intake (g d^−1^) = DMI (g) × [feed lipid content (g kg^−1^ DM) / 1000] × [content of LA/ALNA (g kg^−1^ total FA) in feed / 1000]

Regression equations were developed using residual maximum likelihood analysis, similar to previous studies, so that the potential random effects of experimental period and cow, indicated as significant according to changes in deviance, could be accounted for.^24^ Linear regression equations where the response variables were transfer efficiencies of dietary LA/ALNA to milk and the explanatory variable was WC intake (expressed as kg DM d^−1^) were developed. An approximate *R* ^2^ (pseudo correlation coefficient) was calculated as the squared correlation of the response and the fitted values, to represent the amount of the variability explained.

## RESULTS

### Feed intakes and milk yield and basic composition

The effect of dietary treatment (Control, Low‐WC, High‐WC) on feed intakes was not significant for intakes of total DM (20.5, 20.9, 20.2 kg d^−1^, respectively) and total concentrates (8.3, 8.3, 7.9 kg d^−1^, respectively), and the forage:concentrate ratio of the offered DM was the same throughout the experiment. Significant treatment differences in intake of individual concentrate ingredients were observed but the numerical values of the differences were small (34–850 g of intake, which represented 2–41 g kg^−1^ DMI) (Table 2). As a result of the experimental design, there was a significant effect of dietary treatment (Control, Low‐WC, High‐WC) on intakes of grass (12.2, 8.2, 4.0 kg d^−1^, respectively) and WC (0, 4.4, 8.3 kg d^−1^, respectively). Dietary treatment (Control, Low‐WC, High‐WC) did not significantly affect milk yield (30.5, 31.4, 30.0 kg d^−1^, respectively), and milk fat (37.7, 38.9, 38.9 g kg^−1^, respectively), protein (34.6, 35.0, 33.1 g kg^−1^, respectively) and lactose (47.9, 47.6, 47.5 g kg^−1^, respectively) concentrations.

**Table 2:** Main effect means ± SE and ANOVA P‐values for the effects of dietary treatment on concentrate feed allocation^a^ over the three experimental periods of the feeding trial

Parameters assessed (kg DM per cow d^−1^)	Dietary treatment^b^			ANOVA *P*‐value c
	Control	Low‐WC	High‐WC	
	(*n* = 9)	(*n* = 9)	(*n* = 9)	
Barley	1.7 ± 0.13C	2.0 ± 0.08B	2.3 ± 0.07A	**
Maize grain	1.3 ± 0.09B	1.5 ± 0.05A	1.6 ± 0.03A	**
Wheat feed d	1.3 ± 0.04	1.4 ± 0.04	1.4 ± 0.03	ns
Soya hulls	1.1 ± 0.05B	1.2 ± 0.04B	1.3 ± 0.03A	**
Sugar beet pulp	0.6 ± 0.04A	0.5 ± 0.02B	0.4 ± 0.01C	***
Soybean meal	1.0 ± 0.15A	0.6 ± 0.06B	0.1 ± 0.04C	***
Molasses	0.3 ± 0.01B	0.3 ± 0.01B	0.4 ± 0.01A	*
Pure palm oil	0.2 ± 0.01B	0.2 ± 0.01B	0.2 ± 0.01A	**
Extracted rapeseed meal	0.5 ± 0.08A	0.3 ± 0.03B	0.1 ± 0.02C	***
Limestone flour	0.07 ± 0.005B	0.08 ± 0.005B	0.11 ± 0.006A	***
Salt	0.07 ± 0.002	0.07 ± 0.002	0.07 ± 0.002	ns
Calcined magnesite e	0.05 ± 0.002	0.05 ± 0.002	0.05 ± 0.001	†
Trace elements/vitamins f	0.03 ± 0.001	0.03 ± 0.001	0.03 ± 0.001	ns

DM, dry matter; WC, white clover.

a Daily allocation of concentrates has been developed following estimates of the supply of crude protein from forage (by real‐time assessments of contents of DM and crude protein and feed intake), by combining three types of concentrate pellets with contrasting crude protein contents, made up of different concentrations of similar ingredients. Concentrate feeding aimed to meet energy and protein requirements of the cows, and provide iso‐nitrogenous diets at a constant forage:concentrate ratio of 60:40 (on a DM basis).

b Control, 0 g kg^−1^ fresh‐cut WC + 600 g kg^−1^ fresh‐cut grass + 400 g kg^−1^ concentrate; Low‐WC, 200 g kg^−1^ fresh‐cut WC + 400 g kg^−1^ fresh‐cut grass + 400 g kg^−1^ concentrate; High‐WC, 400 g kg^−1^ fresh‐cut WC + 200 g kg^−1^ fresh‐cut grass + 400 g kg^−1^ concentrate (on a DM basis).

c Significance was declared at: ***P < 0.001, **P < 0.01, *P < 0.05, ^†^0.05 ≤ *P* < 0.10 (trend), ns *P* ≥ 0.10 (non‐significant). Means for dietary treatments within rows and different upper‐case letters are significantly different (*P* < 0.05) according to Fisher's least significant difference test.

d Wheat flour by‐product containing less than 120 g kg^−1^ fibre on a DM basis.

e Produced from the raw material magnesium oxide containing 920 g kg^−1^ MgO.

f Trace elements and vitamins consisted of (kg^−1^): 25 IU vitamin E, 5 mg I, 0.6 mg Se, 30 mg Cu, 50 mg Mn, 100 mg Zn, 9000 IU vitamin A, 2000 IU vitamin D3.

### Milk fatty acid composition

**Figure A1:**
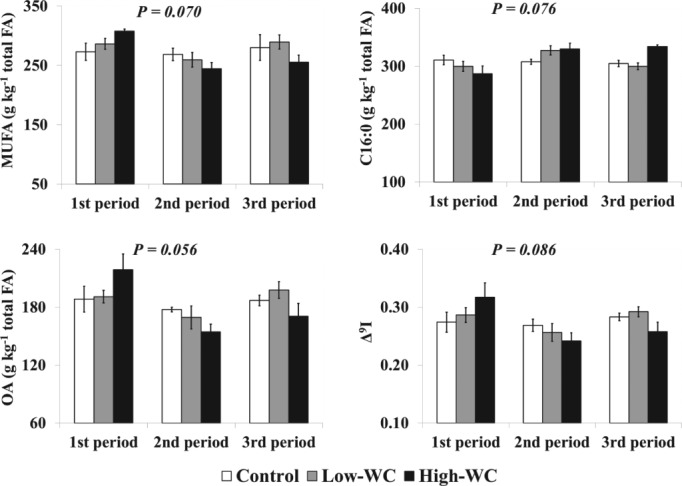
Interaction means ± SE (shown as error bars) for the effects of the interaction between dietary treatment (white clover (WC) offered on a dry matter basis (Control, 0 g kg^−1^, white bars; Low‐WC, 200 g kg^−1^, grey bars; High‐WC, 400 g kg^−1^, black bars) and experimental period on the milk concentrations of monounsaturated fatty acids (MUFA), C16:0, c9 C18:1 (OA, oleic acid) and Δ^9^‐desaturase activity index (Δ^9^I), over the three experimental periods of the feeding trial. P represents the ANOVA P‐value for the interaction.

There was a significant effect of dietary treatment on milk fat concentrations of total PUFA, n‐3 PUFA, n‐6 PUFA, LA and ALNA (Table 3). Cows consuming the Control diet produced milk with less PUFA than cows consuming Low‐WC (−8.8%) and High‐WC (−10.9%) diets (Table 3). Concentrations of n‐3 PUFA, n‐6 PUFA, LA and ALNA in milk fat were increased with increasing contribution of WC in cow diets (+12.2%, +12.9%, +14.1% and + 21.1%, respectively, for Low‐WC, and + 23.4%, +25.8%, +28.9% and 42.5%, respectively, for High‐WC, when compared with Control) (Table 3). Although ANOVA showed a significant effect of dietary treatment on milk fat C12:0 concentration, Fischer's least significance difference test indicated no significant difference between the means (Table 3). Other FA concentrations in milk fat were not significantly affected by diet (Table 3). The interaction between dietary treatment and experimental period was not significant for milk individual FA and FA groups presented in Table 3. However, there was a tendency for an interaction between dietary treatment and experimental period for MUFA, C16:0, OA and Δ^9^I (Appendix Figure A1). The means, SE and ANOVA *P*‐values for the effects of dietary treatment and experimental period, and the ANOVA *P*‐values of their interaction, on the full FA profile of milk fat from cows in the feeding trial are presented in the Appendix (Table A1).

**Table 3:** Main effect means ± SE and ANOVA P‐values for the effects of dietary treatment on nutritionally relevant fatty acid groups and individual fatty acids of milk, over the three experimental periods of the feeding trial

Parameters assessed	Dietary treatment^a^			ANOVA *P*‐value^b^
	Control	Low‐WC	High‐WC	
	(*n* = 9)	(*n* = 9)	(*n* = 9)	
FA groups (g kg^−1^ FA)				
SFA^c^	682 ± 10.2	673 ± 9.4	681 ± 10.8	ns
MUFA^d^	274 ± 8.2	278 ± 7.4	269 ± 10.8	ns
PUFA^e^	44.3 ± 2.45B	48.6 ± 2.33A	49.7 ± 1.39A	*
n‐3^f^	11.5 ± 0.48C	12.9 ± 0.510B	14.2 ± 0.39A	***
n‐6^g^	15.5 ± 0.62C	17.5 ± 0.73B	19.5 ± 0.48A	***
n‐3/n‐6	0.75 ± 0.031	0.74 ± 0.026	0.73 ± 0.021	ns
Individual FA (g kg^−1^ FA)				
C12:0	35.1 ± 1.50A	33.0 ± 1.32A	34.2 ± 2.18A	*
C14:0	116 ± 3.4	113 ± 1.7	113 ± 4.2	ns
C16:0	308 ± 5.2	309 ± 6.0	317 ± 7.9	ns
C18:0	105 ± 4.4	103 ± 4.5	100 ± 5.2	ns
VA	23.9 ± 1.52	24.1 ± 0.85	21.2 ± 1.86	ns
OA	184 ± 6.6	186 ± 6.0	181 ± 10.8	ns
LA	12.8 ± 0.51C	14.6 ± 0.60B	16.5 ± 0.44A	***
RA	10.9 ± 0.92	11.5 ± 0.84	10.0 ± 0.82	ns
ALNA	5.39 ± 0.268C	6.53 ± 0.282B	7.68 ± 0.414A	***
EPA	0.72 ± 0.095	0.64 ± 0.037	0.72 ± 0.038	ns
DPA	1.14 ± 0.130	0.94 ± 0.097	1.17 ± 0.115	ns
FA indices				
Human health‐related				
AI h	2.56 ± 0.127	2.45 ± 0.105	2.56 ± 0.161	ns
TI i	2.86 ± 0.126	2.73 ± 0.118	2.75 ± 0.113	ns
Δ^9^‐Desaturase activity				
Δ^9^I j				ns
C14:1/C14:0	0.07 ± 0.004	0.07 ± 0.004	0.08 ± 0.004	ns
C16:1/C16:0	0.03 ± 0.003	0.03 ± 0.003	0.04 ± 0.002	ns
OA/C18:0	1.78 ± 0.110	1.84 ± 0.105	1.83 ± 0.082	ns
RA/VA	0.46 ± 0.037	0.47 ± 0.028	0.47 ± 0.021	ns

WC, white clover; FA, fatty acid; VA, vaccenic acid; OA, oleic acid; LA, linoleic acid; RA, rumenic acid; ALNA, α‐linolenic acid; EPA, eicosapentaenoic acid; DPA, docosapentaenoic acid; AI, atherogenicity index; TI, thrombogenicity index.

a Control, 0 g kg^−1^ fresh‐cut WC + 600 g kg^−1^ fresh‐cut grass + 400 g kg^−1^ concentrate; Low‐WC, 200 g kg^−1^ fresh‐cut WC + 400 g kg^−1^ fresh‐cut grass + 400 g kg^−1^ concentrate; High‐WC, 400 g kg^−1^ fresh‐cut WC + 200 g kg^−1^ fresh‐cut grass + 400 g kg^−1^ concentrate (on a DM basis).

b Significance was declared at: ***P < 0.001, **P < 0.01, *P < 0.05, ^†^0.05 ≤ *P* < 0.10 (trend), ns *P* ≥ 0.10 (non‐significant). Means for dietary treatments within rows and with different upper‐case letters are significantly different (*P* < 0.05) according to Fisher's least significant difference test.

c SFA: C4:0, C5:0, C6:0, C7:0, C8:0, C9:0, C10:0, C11:0, C12:0, C13:0, C13:0 *iso*, C13:0 *anteiso*, C13:0, C14:0 *iso*, C14:0, C15:0 *anteiso*, C15:0, C16:0 *iso*, C16:0, C17:0 *iso*, C17:0, C18:0 *iso*, C18:0, C20:0, C22:0, C24:0.

d MUFA: *c*9 C10:1, *c*10 C11:1, *c*9 C12:1, *c*9 C13:1, *t*9 14:1, *c*9 C14:1, *c*10 C15:1, *t*7 + *t*8 C16:1, *t*9 C16:1, *t*11 + *t*12 + *t*13 C16:1, *c*9 C16:1 (coelutes with C17:0 *anteiso*), *c*11 C16:1, *c*13 C16:1, *t*10 C17:1, *c*9 C17:1, *t*4 C18:1, *t*5 C18:1, *t*6 + *t*7 + *t*8 C18:1, *t*9 C18:1, *t*10 C18:1, *t*11 C18:1 (VA), *c*6 + *t*12 C18:1, *c*9 C18:1 (OA), *t*15 C18:1, *c*11 C18:1, *c*12 C18:1, *c*13 C18:1, *t*16 + *c*14 C18:1, *c*15 C18:1 (coelutes with C19:0), *c*16 C18:1, *c*5 C20:1, *c*8 C20:1, *c*11 C20:1, *c*13 C22:1, *c*15 C24:1.

e PUFA: *t*11 *t*15 C18:2, *t*9 *t*12 C18:2, *c*9*t*13 C18:2, *c*10*t*14 C18:2, *c*9*t*14 C18:2, *c*9*t*12 C18:2, *t*9*c*12 C18:2, *t*11*c*15 C18:2, *c*9*c*12 C18:2 (LA), *t*12*c*15 C18:2 (coelutes with *c*9 C19:1), *c*6*c*9*c*12 C18:3, *c*9*c*12*c*15 C18:3 (ALNA), *c*9*c*11 C18:2 conjugated (RA) (coelutes with *t*7*c*9 + *t*8*c*10 + *t*6*c*8 C18:2), other C18:2 conjugated FA of unknown isomerism, *c*11*c*14 C20:2, *c*8*c*11*c*14 C20:3, *c*11*c*14*c*17 C20:3, *c*5*c*8*c*11*c*14 C20:4, *c*13*c*16 C22:2, *c*5*c*8*c*11*c*14*c*17 C20:5 (EPA), *c*13*c*16*c*19 C22:3, *c*7*c*10*c*13*c*16*c*19 C22:5 (DPA), *c*4*c*7*c*10*c*13*c*16*c*19 C22:6 (DHA).

f n‐3: *t*11 *t*15 C18:2, *t*11*c*15 C18:2, *t*12*c*15 C18:2 (coelutes with *c*9 C19:1), ALNA, *c*11*c*14*c*17 C20:3, EPA, *c*13*c*16*c*19 C22:3, DPA, DHA.

g n‐6: *t*9 *t*12 C18:2, *c*9*t*12 C18:2, *t*9*c*12 C18:2, LA, *c*6*c*9*c*12 C18:3, *c*11*c*14 C20:2, *c*8*c*11*c*14 C20:3, *c*5*c*8*c*11*c*14 C20:4, *c*13*c*16 C22:2, *c*7*c*10*c*13*c*16 C22:4.

h AI: atherogenicity index = (C12:0 + 4 × C14:0 + C16:0) / (MUFA + PUFA), as described in Srednicka‐Tober *et al*.^42^

i TI: thrombogenicity index = (C14:0 + C16:0 + C18:0) / [(0.5 × MUFA) + (0.5 × n‐6) + (3 × n‐3) + (n‐3/n‐6)], as described in Srednicka‐Tober *et al*.^42^

j Δ^9^I: Δ^9^‐desaturase activity index = (*c*9 C14:1 + *c*9 C16:1 + OA + RA)/(*c*9 C14:1 + *c*9 C16:1 + OA + RA + C14:0 + C16:0 + C18:0 + VA), as proposed by Kay *et al*.^43^

### Fatty acid intakes and transfer efficiencies of dietary PUFA to milk

**Figure 1:**
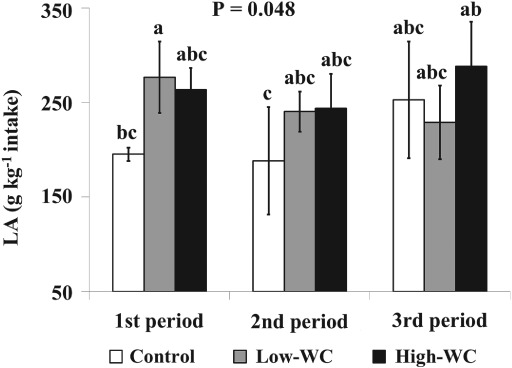
Interaction means ± SE (shown as error bars) for the effects of dietary treatment (white clover (WC) offered on a dry matter basis (Control, 0 g kg^−1^, white bars; Low‐WC, 200 g kg^−1^, grey bars; High‐WC, 400 g kg^−1^, black bars) and experimental period on the transfer efficiency (g kg^−1^ intake) of dietary c9c12 C18:2 (LA, linoleic acid) to milk fat over the three experimental periods of the feeding trial. Bars labelled with different letters are significantly different (P < 0.05) according to Fisher's least significant difference test.

**Figure 2:**
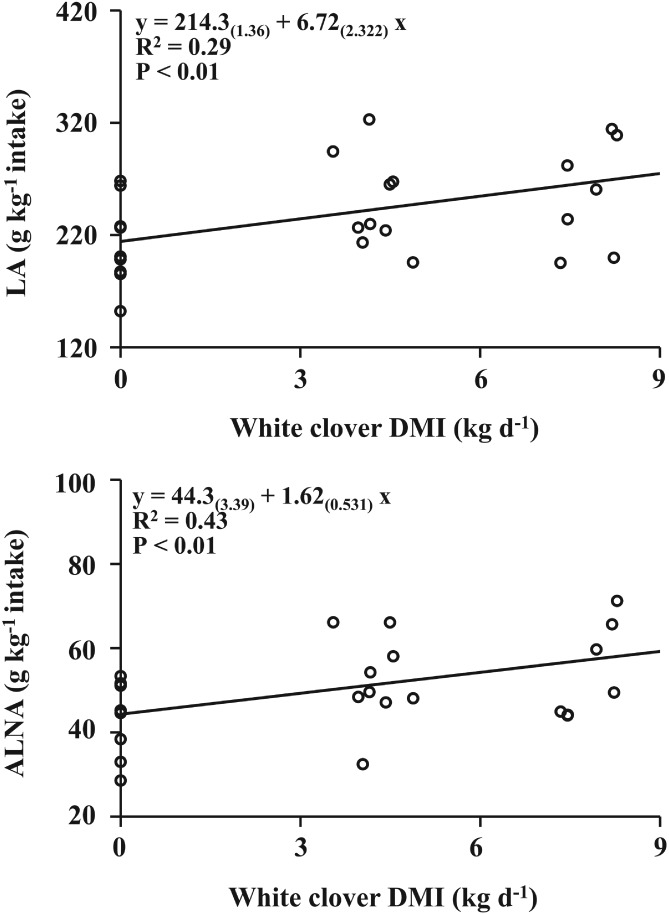
Relationships between white clover dry matter intake (DMI) and transfer efficiencies (g kg^−1^ intake) of c9c12 C18:2 (LA, linoleic acid) and c9c12c15 C18:3 (ALNA, α‐linolenic acid), assessed in individual tie‐stalls with lactating dairy cows over the three experimental periods of the feeding trial. R ^2^ and P represent pseudo‐correlation coefficient and ANOVA P‐value, respectively. Values in parentheses represent SE.

Total FA intake was not different between dietary treatments, but there was a significant effect of dietary treatment on C16:0, C18:0, OA and ALNA intake (Table 4). Cows consuming the Low‐WC diet had a higher C16:0 intake than cows consuming Control and High‐WC diets, but the differences were numerically small (+9 and + 7 g d^−1^, respectively) (Table 4). Cows consuming Control and High‐WC diets had the lowest intakes of C18:0 and OA, respectively, but the differences were numerically small (−1 and − 7 to −9 g d^−1^, respectively), when compared with the other dietary treatments (Table 4). Cows on the Low‐WC and High‐WC diets had higher intakes of ALNA compared with the cows in the Control diet, but differences were numerically small (+13 g d^−1^). When transfer efficiencies of dietary LA and ALNA to milk were assessed, there was a significant effect of diet for ALNA, with transfer efficiency being higher (+13 g kg^−1^ ALNA intake) for High‐WC compared with Control (Table 4). There was a significant interaction between dietary treatment and experimental period for transfer efficiency of LA (Fig. 1), which was higher (+71.4 g kg^−1^ LA intake) for Low‐WC than Control during the first experimental period, but with no significant difference during the second or third experimental periods. Regression equations indicated a positive correlation between transfer efficiencies of dietary LA and ALNA to milk and dietary WC intake, with explained variation being markedly higher in the case of ALNA (*R* ^2^ = 0.43) than LA (*R* ^2^ = 0.29) (Fig. 2).

**Table 4:** Main effect means ± SE and ANOVA P‐values for the effects of dietary treatment on the intakes of main dietary FA, and transfer efficiency of dietary LA and ALNA to milk, over the three experimental periods of the feeding trial

Parameters assessed	Dietary treatment a			ANOVA *P*‐value b
	Control	Low‐WC	High‐WC	
	(*n* = 9)	(*n* = 9)	(*n* = 9)	
Intakes (g d^−1^)				
C16:0	120 ± 4.6B	129 ± 4.3A	122 ± 2.4B	*
C18:0	12 ± 0.4B	13 ± 0.4A	13 ± 0.3A	**
OA	66 ± 2.7A	64 ± 2.0A	57 ± 1.1B	**
LA	64 ± 3.6	67 ± 1.9	66 ± 1.4	ns
ALNA	130 ± 5.3B	143 ± 6.2A	143 ± 5.8A	*
Total FA	431 ± 14.9	462 ± 14.4	442 ± 11.4	ns
Transfer efficiency (g kg^−1^ intake)				
LA	212 ± 12.7	249 ± 13.8	265 ± 15.8	ns
ALNA	44 ± 3B	52 ± 3.5AB	57 ± 3.8A	*

WC, white clover; FA, fatty acids; OA, oleic acid; LA, linoleic acid; ALNA, α‐linolenic acid.

a Control, 0 g kg^−1^ fresh‐cut WC + 600 g kg^−1^ fresh‐cut grass + 400 g kg^−1^ concentrate; Low‐WC, 200 g kg^−1^ fresh‐cut WC + 400 g kg^−1^ fresh‐cut grass + 400 g kg^−1^ concentrate; High‐WC, 400 g kg^−1^ fresh‐cut WC + 200 g kg^−1^ fresh‐cut grass + 400 g kg^−1^ concentrate (on a DM basis).

b Significance was declared at: ***P < 0.001, **P < 0.01, *P < 0.05, ^†^0.05 ≤ *P* < 0.10 (trend), ns *P* ≥ 0.10 (non‐significant). Means for dietary treatments within rows and with different upper‐case letters are significantly different (*P* < 0.05) according to Fisher's least significant difference test.

## DISCUSSION

### Milk yield and basic composition

The effect of grazing WC‐ rather than grass‐based swards is inconsistent, with previous studies reporting higher,^11^, ^12^, ^15^ lower^14^ or similar^13^ milk yield. In studies where milk yield increased with WC consumption, higher yields were attributed to increased DMI and improved nutritive/feeding value of the pasture when WC was fed.^11^, ^12^, ^15^ Contradictory results between previous studies may be explained by differences in DMI, because higher milk yields resulting from WC‐based diets were only observed when cows grazed *ad libitum* and/or higher quantities of WC than grass.^13^ In the current study, although cows were fed *ad libitum* and the High‐WC treatment contained more WC than grass, substitution of fresh‐cut grass with fresh‐cut WC did not have an effect on milk yield. This may be explained by the similar DMI across treatments and the combination of good‐quality forages and high concentrate supply (400 g kg^−1^ DMI), which ensured adequate energy and protein provision to meet cow requirements.

In previous grazing studies,^13^, ^14^ partially replacing grass in cow diets with WC did not affect milk fat concentrations, and this was also observed in the present work. However, an increased contribution of WC in pasture reduced milk fat content in other grazing studies.^11^, ^12^, ^15^ This may be attributed to WC having a higher rate of passage through the rumen than grass,^12^, ^46^ which may reduce rumen fermentation of the diet and therefore rumen acetate and butyrate synthesis, which are substrates of milk fat synthesis by the mammary gland.^47^

The lack of dietary effect on milk protein content reported in this study is in agreement with previous grazing studies.^11^, ^12^, ^13^ This may be explained by the sufficient energy supply across diets to meet cow requirements, due to good‐quality forage and a high contribution of concentrates (400 g kg^−1^ DMI), and the fact that N intake under grazing or zero‐grazing conditions was substantially higher than cow requirements for optimum milk protein production.^12^, ^13^

### Milk fatty acid composition

#### Saturated fatty acids

The lack of dietary treatment effect on milk fat concentrations of total SFA, and the individual SFA C12:0, C14:0 and C16:0, which are assumed undesirable in human diets,^17^ is consistent with previous studies under grazing conditions.^15^ Most milk C12:0 and C14:0 is endogenously synthesized by the mammary gland using acetate and butyrate as substrates,^48^ but dietary effects on their milk concentrations may not be as extensive as for longer‐chain FA.^49^ The finding that replacing dietary grass with WC did not influence C16:0 concentrations in milk is in agreement with previous studies with grazed forages.^15^ Approximately half of milk C16:0 originates from endogenous synthesis by the mammary gland, and therefore its concentrations can be influenced by the diet, either directly or by influencing the ruminal production of substrates for the subsequent *de novo* synthesis in the mammary gland.^48^, ^49^ Increased fresh forage intake in the total diet may reduce milk concentrations of C16:0 and total SFA, but the effect of forage species is not as pronounced.^15^, ^50^, ^51^, ^52^ Therefore, the similar dietary forage:concentrate ratio across dietary treatments in the present study may explain the lack of effects on total and individual SFA concentrations in milk. Concentrations of C18:0 (which has a rather neutral effect on human health)^17^ were not affected by WC inclusion, in agreement with previous studies with grazing cows.^15^

#### Monounsaturated fatty acids

Substituting fresh‐cut grass with fresh‐cut WC in cow diets did not influence milk fat MUFA concentrations, in agreement with grazing studies.^15^ The concentrations of the nutritionally desirable OA^17^, ^18^ may be affected by diet and extent of rumen biohydrogenation (RBH), but also by animal genetics, because a proportion is synthesized in the mammary gland from C18:0 by mammary Δ^9^‐desaturase.^53^, ^54^ The relatively small numerical differences on OA intake from the different dietary treatments (less than 9 g d^−1^ between Control and High‐WC treatments) and the lack of dietary impact on Δ^9^‐desaturase activity, as indicated by Δ^9^I and OA/C18:0 ratio, in the present study, may explain the lack of effect.

The main *trans* MUFA in milk fat is *t*11 C18:1 (VA, vaccenic acid) which is produced in the rumen as an intermediary of the RBH of dietary PUFA, and in particular LA and ALNA.^49^, ^53^ The effect of forage conservation (fresh *vs*. ensiled) is more pronounced on milk VA concentrations than forage species, as reported in previous multivariate analyses.^44^, ^50^, ^52^ In the present study, milk fat VA concentrations were not affected by dietary treatment despite the slightly higher ALNA intakes observed with higher WC intake. This may indicate that a smaller proportion of dietary ALNA was hydrogenated to VA in the rumen when cows were fed the WC‐containing diets. Although fresh grass consumption (either grazed or zero‐grazed) may result in an increase in milk VA concentrations,^50^, ^51^, ^52^, ^55^ the effect of grazing was more pronounced when compared with zero‐grazing practices (such as those used in the current work) in other studies.^56^

#### Polyunsaturated fatty acids

In the present study, cows consuming Low‐WC and High‐WC diets produced milk with increased fat concentrations of the nutritionally essential LA and ALNA.^17^, ^18^, ^21^ The simultaneous increase in milk LA and ALNA concentrations with increasing WC intake resulted in a similar n‐3:n‐6 ratio across the dietary treatments. The increased concentration of ALNA in milk with WC‐based diets was consistent with previous work with grazing cows.^15^ In previous research,^56^ zero‐grazing practices resulted in a 30% reduction in milk ALNA concentration when compared with grazing, because of the loss of ALNA during wilting. Although forage‐handling practices in the current study minimized potential wilting, the potential effect on milk from grazing cows receiving the same amounts of WC may be even higher.

In the present study, diets were formulated in order to meet energy and protein requirements of cows under a constant forage:concentrate ratio (on a DM basis), by also minimizing the potential difference on dietary PUFA supply between the dietary treatments. Therefore, the difference between the lowest and highest intakes were only 1.5 g d^−1^ for LA and 13 g d^−1^ for ALNA. Interestingly, increased milk LA and ALNA concentrations were observed in WC‐based treatments despite the similar intakes of LA and ALNA. These FA cannot be endogenously synthesized and, given that cows were in mid‐lactation and did not experience negative energy balance at any time during the experiment, adipose tissue lipid mobilization would be limited.^49^ Therefore, milk LA and ALNA could be expected to be predominantly of dietary origin. The higher concentrations of LA and ALNA in milk fat following WC‐based diets may be explained by higher transfer efficiencies from diet to milk. For ALNA, this was true throughout the study, as there was an increase of + 8 and + 13 g kg^−1^ ALNA intake, for Low‐WC (range 32–66 g kg^−1^) and High‐WC diets (range 44–72 g kg^−1^), respectively, when compared with Control diet (range 29–53 g kg^−1^). For LA, an effect was observed only during the first experimental period, as there was an increase of +82 g kg^−1^ LA intake for the Low‐WC diet (range 195–323 g kg^−1^) when compared with the Control diet (range 152–268 g kg^−1^).

Previous studies investigating *in vivo* nutrient rumen degradability of fresh‐cut forages have reported higher DM degradation rates in WC when compared with grass.^46^ In addition, the Control diet had higher NDF and ADF contents (416 and 203 g kg^−1^ DM, respectively), than the Low‐WC (390 and 200 g kg^−1^ DM, respectively) and High‐WC (362 and 193 g kg^−1^ DM, respectively) diets. Therefore, WC possibly demonstrated higher rumen passage rates than grass, because less‐degradable feed particles high in cellulose are retained in the rumen for longer.^47^ This may have reduced exposure of dietary PUFA to rumen microorganisms, thus resulting in lower RBH rates of LA and ALNA and greater amounts being available for absorption in the small intestine. In addition, despite slightly higher ALNA intakes for the High‐WC diet, milk VA and RA concentrations were numerically slightly lower compared with cows fed Control. This indicates a potential slower rate, although not statistically significant, of RBH for ALNA. This is also supported by the significantly higher output of ALNA and LA in milk from the Low‐Clover (7.9 and 17.6 g d^−1^) and High‐Clover (8.7 and 18.6 g d^−1^) diets when compared with the Control milk (6.0 and 14.2 g d^−1^), while the effect of treatment on milk VA and RA outputs was not statistically significant (results not shown).

In pasture‐based dairy systems, WC is used because of its potential to reduce usage of imported protein and inorganic N fertilizer, while also increasing milk production in some cases.^7^, ^8^, ^9^ The increased n‐3 PUFA concentrations in milk may be considered an additional benefit to the overall sustainability of this practice rather than a dietary strategy to achieve extensive changes in milk FA profile, because the influence on milk n‐3 PUFA was relatively low. Under the reported average consumption of milk in the UK (per person, 767 g d^−1^),^57^ and according to the findings of this research, a switch from milk from Control‐fed cows to milk from High‐WC‐fed cows will offer an increase in dietary ALNA intake of 68 mg d^−1^. Under the current daily reference values for ALNA intake in the UK (>0.2% of total energy intake,^58^ and assuming an average energy intake for men and women at 2500 and 2000 kcal d^−1^, respectively, this would increase the contribution of milk fat in ALNA intake to men and women, in the UK, from 26% to 38% and from 33% to 48%, respectively. However, conclusions regarding the influence of these changes on human health cannot be drawn by the present study. In comparison with previously reported ALNA concentrations in UK conventional retail milk (4.4 g kg^−1^ total FA),^55^ consuming milk from cows fed High‐WC diet in this study would offer an increase in (i) dietary ALNA intake of 103 mg d^−1^ and (ii) the contributions of milk fat to ALNA dietary reference values in men and women, in the UK, from 20% to 38% and from 25% to 48%, respectively. These differences, however, may be explained by the relatively high fresh‐cut forage intake (600 g kg^−1^ DMI) in the diets of the current study compared with the influence of WC alone.

Another nutritionally beneficial PUFA in milk is RA,^17^, ^18^, ^22^ which is unique to ruminant products as it is synthesized primarily in the mammary gland, using VA as a substrate, by the Δ^9^‐desaturase enzyme.^59^ In studies where cows grazed WC‐based or grass‐based swards, an effect on milk RA concentrations was not observed,^15^ in agreement with the present study. The proportion of fresh forage in the diet has been a more pronounced driver for milk RA concentrations than forage species, in previous multivariate redundancy analyses.^51^, ^52^, ^60^ In the current study, constant dietary forage:concentrate ratio across dietary treatments meant that the effect of WC intake on milk RA concentrations was not significant, despite slightly higher intakes of ALNA and LA, which are substrates for VA synthesis in the rumen.^49^, ^53^ Also, zero‐grazing practices used in the current study may not have as strong an impact on milk RA concentrations as grazing the same amounts of forage at pasture; this has been previously demonstrated as a potential result of the loss of ALNA during wilting in zero‐grazing practices.^56^

Δ^9^‐desaturase activity indices, dietary intakes of FA and concentrations of short‐ and medium‐chain FA were similar between treatments. This may indicate that observed changes in milk FA profile, as a result of different dietary treatments, may be largely explained by changes in the rumen environment and RBH than from enzyme activity in the mammary gland. Previous research involving WC‐based diets indicated that any dietary effect on the activity of Δ^9^‐desaturase is possibly driven by the amounts of fresh forage intake rather than the relative contribution of grass and WC.^15^

Very‐long‐chain PUFA associated with positive implications in human health, such as EPA and DPA,^17^, ^18^, ^20^, ^23^ as well as indices associated with the atherogenicity and thrombogenicity potential of milk fat, have not been influenced by dietary WC, in agreement with previous research with grazed WC.^15^

## CONCLUSION

This study is the first to report an increase in transfer efficiency of dietary LA (in one experimental period) and ALNA (throughout the study) to milk, by substituting fresh‐cut grass with fresh‐cut white clover in dairy cow diets. This effect may be due to a reduced rate of rumen biohydrogenation of LA and ALNA when fresh‐cut white clover was used. Including white clover in grazing or zero‐grazed swards can offer a sustainable option to increase nutritionally beneficial n‐3 PUFA concentrations in milk from pasture‐based systems, without implications on productivity and milk basic composition. The desirable outcomes may be enhanced with increasing dietary contribution of white clover (up to 400 g kg^−1^ DMI) but it remains unclear whether these effects on milk composition would have a significant impact upon human health. The relatively low impact of the dietary treatments, under a constant forage:concentrate ratio (on a DM basis), on the overall milk FA profile indicates that increasing fresh forage intake may be a more effective strategy to increase nutritionally beneficial FA and reduce SFA in milk, as shown by other studies in different dairy production systems. The increased n‐3 PUFA concentrations in milk from cows fed WC‐based diets can be seen as an additional benefit to the overall sustainability of including white clover in dairy cow diets, rather than a method to extensively modify milk FA profiles in pasture‐based and zero‐grazing systems.

## Appendix

**Table A1:** Main effect means ± SE and ANOVA P‐values for the effects of dietary treatment and experimental period on the concentrations of all identified individual fatty acids (FA) in milk from cows in the feeding trial

FA (g kg^−1^ total FA)	Dietary treatment (D) a			Experimental period (P)			ANOVA *P*‐values b		
	Control (*n* = 9)	Low‐WC (*n* = 9)	High‐WC (*n* = 9)	1st (*n* = 9)	2nd (*n* = 9)	3rd (*n* = 9)	D	P	D x P
C4:0	23.3 ± 0.90	23.2 ± 0.52	24.0 ± 0.68	25.1 ± 0.64A	23.0 ± 0.49B	22.4 ± 0.67B	†	**	ns
C5:0	0.132 ± 0.0386	0.082 ± 0.0343	0.103 ± 0.0351	0.050 ± 0.0265B	0.118 ± 0.0271AB	0.149 ± 0.0444A	ns	*	ns
C6:0	16.0 ± 0.52	15.8 ± 0.37	16.2 ± 0.29	16.4 ± 0.36A	16.3 ± 0.34A	15.3 ± 0.42B	ns	*	ns
C7:0	0.178 ± 0.0481	0.141 ± 0.0427	0.137 ± 0.0343	0.085 ± 0.0390	0.181 ± 0.0315	0.189 ± 0.0457	ns	†	ns
C8:0	10.5 ± 0.29	10.1 ± 0.24	10.4 ± 0.29	10.1 ± 0.24B	10.7 ± 0.26A	10.2 ± 0.29B	ns	*	ns
C9:0	0.289 ± 0.0499	0.241 ± 0.0334	0.235 ± 0.0332	0.194 ± 0.0337B	0.281 ± 0.0277A	0.289 ± 0.0483A	ns	*	ns
C10:0	27.9 ± 1.05	26.5 ± 0.88	27.3 ± 1.36	25.4 ± 0.77C	29.2 ± 1.01A	27.1 ± 1.16B	†	**	ns
*c*9 C10:1	2.35 ± 0.151	2.38 ± 0.086	2.50 ± 0.152	2.23 ± 0.099	2.44 ± 0.155	2.56 ± 0.119	ns	†	ns
C11:0	0.580 ± 0.1005	0.478 ± 0.0625	0.463 ± 0.0661	0.394 ± 0.0616	0.556 ± 0.0557	0.570 ± 0.1004	†	†	ns
C12:0	35.1 ± 1.5A	33.0 ± 1.3A	34.2 ± 2.2A	30.5 ± 1.07C	37.3 ± 1.43A	34.4 ± 1.7B	*	**	ns
C13:0 *iso*	0.371 ± 0.0337	0.334 ± 0.0301	0.337 ± 0.0204	0.333 ± 0.0299	0.336 ± 0.0297	0.373 ± 0.0257	ns	ns	ns
C13:0 *anteiso*	0.799 ± 0.0573	0.732 ± 0.0290	0.771 ± 0.0747	0.602 ± 0.0385C	0.813 ± 0.0199B	0.888 ± 0.0510A	†	**	†
*c*9 C12:1	0.867 ± 0.0412	0.796 ± 0.0473	0.846 ± 0.0748	0.708 ± 0.0390B	0.932 ± 0.0470A	0.870 ± 0.0527A	ns	**	ns
C13:0	0.943 ± 0.1362	0.819 ± 0.0963	0.764 ± 0.0831	0.714 ± 0.0916	0.894 ± 0.0806	0.918 ± 0.1372	†	ns	ns
C14:0 *iso*	0.791 ± 0.0381	0.757 ± 0.0283	0.707 ± 0.0287	0.674 ± 0.0215B	0.762 ± 0.0328A	0.820 ± 0.0249A	†	*	ns
c*9* C13:1	0.606 ± 0.0619	0.609 ± 0.0562	0.641 ± 0.0538	0.484 ± 0.0244C	0.601 ± 0.0554B	0.771 ± 0.0330A	ns	**	ns
C14:0	116 ± 3.4	113 ± 1.7	113 ± 4.2	107 ± 2.4C	121 ± 2.7A	114 ± 3.0B	ns	***	ns
t*9* C14:1	2.35 ± 0.068A	2.20 ± 0.037B	2.00 ± 0.033C	2.21 ± 0.058	2.18 ± 0.086	2.16 ± 0.063	***	ns	ns
C15:0 *anteiso*	5.03 ± 0.165	5.04 ± 0.190	4.52 ± 0.213	4.73 ± 0.189	5.01 ± 0.125	4.85 ± 0.274	†	ns	ns
*c*9 C14:1	8.55 ± 0.426	8.14 ± 0.369	8.61 ± 0.600	7.64 ± 0.465B	8.81 ± 0.375A	8.84 ± 0.468A	ns	*	ns
C15:0	10.6 ± 0.81	10.4 ± 0.66	10.0 ± 0.49	9.6 ± 0.67	10.5 ± 0.44	10.8 ± 0.77	ns	†	ns
C16:0 *iso*	1.89 ± 0.064	1.82 ± 0.034	1.72 ± 0.037	1.82 ± 0.033	1.82 ± 0.072	1.79 ± 0.044	†	ns	ns
*c*10 C15:1	0.056 ± 0.0377	0.072 ± 0.0394	0.029 ± 0.0220	0.020 ± 0.0138	0.088 ± 0.0452	0.049 ± 0.0325	ns	ns	ns
C16:0	308 ± 5.2	309 ± 6.0	317 ± 7.9	299 ± 4.6B	322 ± 5.2A	313 ± 7.2A	ns	**	†
*t*6 + *t*7 + *t*8 C16:1	0.677 ± 0.0901	0.616 ± 0.0259	0.651 ± 0.0525	0.717 ± 0.0773	0.623 ± 0.0517	0.604 ± 0.0473	ns	ns	ns
*t*9 C16:1	1.22 ± 0.063	1.26 ± 0.080	1.16 ± 0.109	1.27 ± 0.082	1.28 ± 0.084	1.08 ± 0.077	ns	ns	ns
C17:0 *iso*	4.19 ± 0.128	4.11 ± 0.163	3.84 ± 0.185	4.51 ± 0.082A	3.87 ± 0.122B	3.76 ± 0.152B	†	**	ns
*t*11 + *t*12 + *t*13 C16:1	1.72 ± 0.144	1.64 ± 0.156	1.68 ± 0.113	1.70 ± 0.140	1.45 ± 0.143	1.90 ± 0.077	ns	ns	ns
*c*9 C16:1 + C17 *anteiso*	11.9 ± 0.42B	11.7 ± 0.53B	12.8 ± 0.63A	13.0 ± 0.47	11.8 ± 0.47	11.8 ± 0.62	*	†	ns
*c*11 C16:1	4.27 ± 0.522	4.77 ± 0.148	3.96 ± 0.571	4.94 ± 0.113	4.65 ± 0.115	3.42 ± 0.688	†	†	*
*c*13 C16:1	2.97 ± 0.126	3.02 ± 0.124	2.91 ± 0.156	2.73 ± 0.136B	3.09 ± 0.097A	3.08 ± 0.135A	ns	*	ns
C17:0	4.99 ± 0.203	5.07 ± 0.207	5.23 ± 0.186	5.46 ± 0.163	4.96 ± 0.125	4.87 ± 0.235	ns	†	ns
C18:0 *iso*	1.22 ± 0.072A	1.09 ± 0.043AB	0.97 ± 0.062B	1.24 ± 0.044A	0.94 ± 0.047C	1.09 ± 0.070B	**	**	ns
*c*9 C17:1	1.90 ± 0.157	1.76 ± 0.167	2.04 ± 0.206	2.40 ± 0.142A	1.64 ± 0.126B	1.65 ± 0.126B	†	**	ns
C18:0	105 ± 4.4	103 ± 4.5	100 ± 5.2	111 ± 4.7A	96 ± 4.4C	101 ± 3.6B	ns	***	ns
*t*4 C18:1	0.145 ± 0.0424	0.158 ± 0.0454	0.173 ± 0.0321	0.217 ± 0.0370	0.114 ± 0.0346	0.145 ± 0.0409	ns	ns	ns
*t*5 C18:1	0.209 ± 0.0305	0.200 ± 0.0469	0.163 ± 0.0410	0.109 ± 0.0323B	0.174 ± 0.0325AB	0.289 ± 0.0279A	ns	*	ns
*t*6 + *t*7 + *t*8 C18:1	3.04 ± 0.239	3.35 ± 0.099	3.04 ± 0.090	3.14 ± 0.086	3.01 ± 0.226	3.28 ± 0.139	ns	ns	ns
t9 C18:1	2.25 ± 0.130	2.28 ± 0.140	2.05 ± 0.116	2.16 ± 0.080	2.24 ± 0.166	2.18 ± 0.141	ns	ns	ns
*t*10 C18:1	5.55 ± 1.046	6.02 ± 0.773	5.04 ± 0.363	4.89 ± 0.751	5.16 ± 0.377	6.56 ± 0.989	ns	ns	ns
*t*11 C18:1	23.9 ± 1.52	24.1 ± 0.85	21.2 ± 1.86	22.0 ± 1.21	25.4 ± 1.10	21.8 ± 1.87	ns	ns	ns
*c*6 + *t*12 C18:1	3.93 ± 0.352	4.30 ± 0.288	4.55 ± 0.181	4.41 ± 0.307	4.01 ± 0.274	4.36 ± 0.288	ns	ns	ns
*c*9 C18:1	184 ± 6.6	186 ± 6.0	181 ± 10.8	199 ± 6.5A	167 ± 5.8C	185 ± 7.6B	ns	**	†
*t*15 C18:1	3.25 ± 0.309	3.78 ± 0.237	3.40 ± 0.172	3.38 ± 0.290	3.32 ± 0.202	3.73 ± 0.251	ns	ns	ns
*c*11 C18:1	4.11 ± 0.396	3.97 ± 0.289	4.07 ± 0.484	4.91 ± 0.405A	3.48 ± 0.325B	3.75 ± 0.251B	ns	**	ns
*c*12 C18:1	1.99 ± 0.070B	2.50 ± 0.099A	2.69 ± 0.080A	2.41 ± 0.128	2.31 ± 0.126	2.47 ± 0.139	***	ns	ns
*c*13 C18:1	0.949 ± 0.0786	0.974 ± 0.0568	0.904 ± 0.0561	0.970 ± 0.0587	0.862 ± 0.0397	0.995 ± 0.0812	ns	ns	ns
*t*16 + *c*14 C18:1	4.67 ± 0.361	5.18 ± 0.240	4.87 ± 0.137	4.87 ± 0.264	4.68 ± 0.157	5.17 ± 0.338	ns	ns	ns
*c*15 C18:1 + C19:0	1.06 ± 0.167	1.21 ± 0.202	1.02 ± 0.169	1.38 ± 0.070	0.95 ± 0.206	0.94 ± 0.192	ns	ns	ns
*t*11 *t*15 C18:2	0.329 ± 0.1561	0.302 ± 0.1594	0.344 ± 0.1871	0.014 ± 0.0135	0.533 ± 0.2073	0.428 ± 0.1514	ns	ns	ns
*t*9 *t*12 C18:2	0.211 ± 0.0702	0.268 ± 0.0648	0.155 ± 0.0489	0.103 ± 0.0604B	0.214 ± 0.0427AB	0.318 ± 0.0627A	ns	*	ns
*c*9*t*13 C18:2	2.67 ± 0.591	3.13 ± 0.408	2.73 ± 0.228	2.66 ± 0.392	2.57 ± 0.219	3.30 ± 0.583	ns	ns	ns
*c*10*t*14 C18:2	1.63 ± 0.443	1.12 ± 0.189	0.99 ± 0.088	1.10 ± 0.142	1.30 ± 0.396	1.33 ± 0.296	ns	ns	ns
*c*9*t*14 C18:2	1.07 ± 0.136	1.23 ± 0.121	1.14 ± 0.119	1.20 ± 0.124	1.06 ± 0.091	1.17 ± 0.155	ns	ns	ns
*c*9*t*12 C18:2	0.334 ± 0.0404	0.358 ± 0.0521	0.320 ± 0.0266	0.355 ± 0.0429	0.298 ± 0.0394	0.359 ± 0.0383	ns	ns	ns
*c*16 C18:1	0.734 ± 0.0669	0.795 ± 0.0734	0.810 ± 0.0295	0.786 ± 0.0573	0.688 ± 0.0478	0.866 ± 0.0600	ns	†	ns
*t*9*c*12 C18:2	0.535 ± 0.0334	0.587 ± 0.0593	0.582 ± 0.0467	0.544 ± 0.0616	0.608 ± 0.0430	0.553 ± 0.0328	ns	ns	ns
*t*11*c*15 C18:2	3.22 ± 0.282	3.81 ± 0.365	3.42 ± 0.286	3.18 ± 0.413	3.62 ± 0.237	3.65 ± 0.270	ns	ns	ns
*c*9*c*12 C18:2	12.8 ± 0.51C	14.6 ± 0.60B	16.5 ± 0.44A	15.5 ± 0.75A	14.0 ± 0.79B	14.5 ± 0.60AB	***	*	ns
*t*12*c*15 C18:2 + *c*9 C19:1	0.809 ± 0.0577	0.901 ± 0.0758	0.949 ± 0.0591	0.942 ± 0.0499	0.773 ± 0.0652	0.945 ± 0.0682	ns	†	ns
C20:0	1.27 ± 0.053B	1.40 ± 0.054A	1.36 ± 0.051A	1.38 ± 0.046	1.32 ± 0.052	1.33 ± 0.065	*	ns	ns
*c*6*c*9*c*12 C18:3	0.237 ± 0.0307	0.255 ± 0.0332	0.247 ± 0.0279	0.183 ± 0.0224B	0.230 ± 0.0281B	0.326 ± 0.0146A	ns	**	ns
*c*9*c*12*c*15 C18:3	5.39 ± 0.268C	6.53 ± 0.282B	7.68 ± 0.414A	6.22 ± 0.427	6.36 ± 0.432	7.02 ± 0.487	***	†	ns
*c*11 C20:1	0.426 ± 0.0539B	0.583 ± 0.0330A	0.548 ± 0.0520A	0.633 ± 0.0410A	0.484 ± 0.0470B	0.439 ± 0.0439B	*	*	ns
*c*9*t*11 C18:2	10.9 ± 0.92	11.5 ± 0.84	10.0 ± 0.82	9.4 ± 0.90B	11.8 ± 0.78A	11.1 ± 0.75AB	ns	*	ns
Unknown C18:2 conjugated	0.467 ± 0.0695	0.611 ± 0.0619	0.603 ± 0.0668	0.489 ± 0.0648	0.561 ± 0.0565	0.631 ± 0.0783	ns	ns	ns
Unknown C18:2 conjugated	0.568 ± 0.0742	0.588 ± 0.1053	0.665 ± 0.0961	0.838 ± 0.0586A	0.623 ± 0.0922A	0.360 ± 0.0165B	ns	**	ns
*c*11*c*14 C20:2	0.117 ± 0.0259B	0.135 ± 0.0314AB	0.206 ± 0.0214A	0.144 ± 0.0350	0.185 ± 0.0235	0.130 ± 0.0263	*	ns	ns
C22:0	0.95 ± 0.117	0.93 ± 0.108	1.00 ± 0.131	1.07 ± 0.105A	1.19 ± 0.092A	0.62 ± 0.020B	ns	**	ns
*c*11*c*14*c*17 C20:3	0.194 ± 0.0515	0.229 ± 0.1419	0.307 ± 0.0818	0.432 ± 0.1408	0.219 ± 0.0428	0.079 ± 0.0283	ns	ns	ns
*c*5*c*8*c*11*c*14 C20:4	0.96 ± 0.053	1.03 ± 0.073	1.07 ± 0.045	1.10 ± 0.050	1.05 ± 0.058	0.90 ± 0.046	ns	†	ns
*c*13 C22:1	0.000 ± 0.0000	0.000 ± 0.0000	0.084 ± 0.0844	0.000 ± 0.0000	0.084 ± 0.0844	0.000 ± 0.0000	ns	ns	ns
*c*13*c*16 C22:2	0.029 ± 0.0291B	0.000 ± 0.0000B	0.077 ± 0.0551A	0.000 ± 0.0000	0.000 ± 0.0000	0.106 ± 0.0576	***	ns	***
*c*5*c*8*c*11*c*14*c*17 C20:5	0.718 ± 0.0951	0.640 ± 0.0373	0.717 ± 0.0377	0.738 ± 0.0270	0.669 ± 0.1019	0.668 ± 0.0289	ns	ns	ns
*c*13*c*16*c*19 C22:3	0.089 ± 0.0890	0.025 ± 0.0245	0.021 ± 0.0206	0.025 ± 0.0245	0.110 ± 0.0888	0.000 ± 0.0000	ns	ns	ns
*c*15 C24:1	0.014 ± 0.0144	0.000 ± 0.0000	0.000 ± 0.0000	0.000 ± 0.0000	0.014 ± 0.0144	0.000 ± 0.0000	ns	ns	ns
*c*7*c*10*c*13*c*16 C22:4	0.243 ± 0.0365	0.235 ± 0.0572	0.254 ± 0.0489	0.352 ± 0.0238A	0.287 ± 0.0372A	0.093 ± 0.0226B	ns	***	*
*c*7*c*10*c*13*c*16*c*19 C22:5	1.14 ± 0.130	0.94 ± 0.097	1.17 ± 0.115	1.19 ± 0.157	1.12 ± 0.109	0.94 ± 0.050	ns	ns	ns
*c*4*c*7*c*10*c*13*c*16*c*19 C22:6	0.056 ± 0.0328	0.000 ± 0.0000	0.077 ± 0.0365	0.084 ± 0.0336	0.049 ± 0.0351	0.000 ± 0.0000	ns	†	ns

a Control, 0 g kg^−1^ fresh‐cut WC + 600 g kg^−1^ fresh‐cut grass + 400 g kg^−1^ concentrate; Low‐WC, 200 g kg^−1^ fresh‐cut WC + 400 g kg^−1^ fresh‐cut grass + 400 g kg^−1^ concentrate; High‐WC, 400 g kg^−1^ fresh‐cut WC + 200 g kg^−1^ fresh‐cut grass + 400 g kg^−1^ concentrate (on a DM basis).

b Significance was declared at: ****P* < 0.001, ***P* < 0.01, **P* < 0.05, ^†^0.05 ≤ *P* < 0.10 (trend), ns *P* ≥ 0.10 (non‐significant). Means for dietary treatments within rows, or for different experimental periods within rows and with different upper‐case letters are significantly different (*P* < 0.05) according to Fisher's least significant difference test.
